# Associations of Health App Use and Perceived Effectiveness in People With Cardiovascular Diseases and Diabetes: Population-Based Survey

**DOI:** 10.2196/12179

**Published:** 2019-03-28

**Authors:** Clemens Ernsting, Lena Mareike Stühmann, Stephan U Dombrowski, Jan-Niklas Voigt-Antons, Adelheid Kuhlmey, Paul Gellert

**Affiliations:** 1 Charité - Universitätsmedizin Berlin Institute of Medical Sociology Berlin Germany; 2 University of New Brunswick Faculty of Kinesiology Fredericton, NB Canada; 3 Technische Universität Berlin Quality and Usability Lab Berlin Germany

**Keywords:** mHealth, eHealth, smartphone, telemedicine, health literacy, chronic disease, comorbidity, multimorbidity

## Abstract

**Background:**

Mobile health apps can help to change health-related behaviors and manage chronic conditions in patients with cardiovascular diseases (CVDs) and diabetes mellitus, but a certain level of health literacy and electronic health (eHealth) literacy may be needed.

**Objective:**

The aim of this study was to identify factors associated with mobile health app use in individuals with CVD or diabetes and detect relations with the perceived effectiveness of health apps among app users.

**Methods:**

The study used population-based Web-based survey (N=1500) among Germans, aged 35 years and older, with CVD, diabetes, or both. A total of 3 subgroups were examined: (1) Individuals with CVD (n=1325), (2) Individuals with diabetes (n=681), and (3) Individuals with CVD and diabetes (n=524). Sociodemographics, health behaviors, CVD, diabetes, health and eHealth literacy, characteristics of health app use, and characteristics of apps themselves were assessed by questionnaires. Linear and logistic regression models were applied.

**Results:**

Overall, patterns of factors associated with health app use were comparable in individuals with CVD or diabetes or both. Across subgroups, about every fourth patient reported using apps for health-related purposes, with physical activity and weight loss being the most prominent target behaviors. Health app users were younger, more likely to be female (except in those with CVD and diabetes combined), better educated, and reported more physical activity. App users had higher eHealth literacy than nonusers. Those users who perceived the app to have a greater effectiveness on their health behaviors tended to be more health and eHealth literate and rated the app to use more behavior change techniques (BCTs).

**Conclusions:**

There are health- and literacy-related disparities in the access to health app use among patients with CVD, diabetes, or both, which are relevant to specific health care professionals such as endocrinologists, dieticians, cardiologists, or general practitioners. Apps containing more BCTs had a higher perceived effect on people’s health, and app developers should take the complexity of needs into account. Furthermore, eHealth literacy appears to be a requirement to use health apps successfully, which should be considered in health education strategies to improve health in patients with CVD and diabetes.

## Introduction

### Cardiovascular Diseases and Diabetes

Cardiovascular conditions such as myocardial infarction, stroke, or coronary artery diseases are the main causes of death worldwide [1], and diabetes mellitus is a major risk factor for cardiovascular diseases (CVDs) [2,3]. In a large cohort study from the United Kingdom, for instance, about 18 percent of those with diabetes showed incident CVD over the 5.5 years of observation [4]. However, diabetes itself is a serious disease with substantial health consequences besides cardiovascular events, including end-stage renal diseases, loss of vision, or limb amputations [5]. Over the past decades, the prevalence of diabetes has dramatically increased worldwide, which has been labeled as a diabetes pandemic [6,7]. CVD and diabetes have been considered as concordant chronic comorbidities [8], as they have many risk factors in common, including overweight, smoking, hypertension, and physical inactivity [5,9]. Thus, CVD and diabetes frequently coexist as comorbidities within the same people [10]. Understanding medical management and self-care of (1) CVD, (2) diabetes alone, and (3) CVD and diabetes in combination may improve clinical outcomes and quality of life in people diagnosed with these conditions [8].

CVD and diabetes share common risk factors, most of which can be ameliorated via health behavioral changes [11-14]. Health behavior change is crucial to prevent and treat these chronic conditions [15-17]. As people diagnosed with CVD or diabetes commonly require continuous lifelong treatment, supporting individuals to implement behavior change recommendations is critical to improve the disease management. Mobile health apps are a promising tool to modify behavioral risk factors and support disease management [18-22].

### Mobile Health Apps

Mobile health apps have changed most areas of daily living, including health and diseases management [19]. People now have the opportunity to access information, communicate with others at anyplace and anytime, track relevant behaviors and outcomes over time and location, and receive additional health behavior change input. Evidence from the general population suggests that people with chronic conditions, including CVD and diabetes, more frequently use mobile health apps compared with healthy individuals [23]. However, large proportions of individuals with chronic diseases do not engage in mobile health app use and potentially miss out on the benefits that novel health technologies have to offer [22,24-27]. The barriers to engaging in health apps require further attention, particularly in individuals with existing chronic conditions such as CVD and diabetes.

Evidence from systematic reviews of randomized controlled trials shows positive effects of mobile health apps for diabetes to support improvements in hemoglobin A_1c_ (HbA_1c_) and glycemic control [28-31]. Mobile health apps for CVD, which focus on the modification of cardiovascular risk factors or medication adherence, have the potential to enhance people’s health [32,33]. Although there are studies for those suffering from CVD or diabetes, studies that look into app use of patients with CVD and diabetes combined are needed.

Despite the potential for benefit of mobile apps, disparities in the access and variability in the effectiveness exist. For example, population-based surveys reported that people with low education are less likely [23,27,34] and those who are diagnosed with multiple chronic conditions are more likely to use health apps [23]. Moreover, more frequent engagement with apps has been found to be associated with better perceived effects [35,36]. In addition to app users’ cognitive, health, and engagement factors, characteristics of the apps can influence their effectiveness. Ernsting et al (2017), Webb et al (2010), and Morrissey et al (2016) found that health apps applying specific behavior change techniques (BCTs) such as planning or monitoring were more effective in health promotion [23,37,38].

### Health Literacy and Electronic Health Literacy

Health literacy is one of the key features for successful disease management [39-41]. It is a requirement to access and understand health information and make decisions concerning health care [42]. As mobile technologies have emerged, the concept of electronic health (eHealth) literacy was introduced, which is the ability to use information technology for health [43]. Emerging evidence suggests that general population samples and primary care patients with higher health literacy and eHealth literacy are more likely to use mobile health apps and perceive these to be more effective [23,25,27,43-45]. However, the specific relation of health literacy and eHealth literacy with app use among those with CVD, diabetes, or both has not yet been examined.

### Aims of the Study

Although sociodemographic factors and health literacy and eHealth literacy related with the utilization of health apps among the general population are known, these associations among specific epidemiologically and clinically relevant subgroups, that is, those with CVD, diabetes, and both combined need investigation. There are no studies investigating these subgroups within 1 study.

Thus, the aims of the study were to investigate health literacy and eHealth literacy of app users beyond sociodemographic factors in clinically relevant subgroups and explore the association of these characteristics with the perceived effectiveness of mobile health apps on a participant’s health. More specifically, we aimed for the following:

1. To estimate the utilization of health apps in a population-based sample among specific clinically relevant subgroups, that is, CVD, diabetes, and both combined.
2. To investigate which factors (ie, age, gender, education, health behaviors, disease burden, health literacy, eHealth literacy, and wearable use) are associated with health app use—separately for CVD, diabetes, and both combined.
3. To investigate which factors (ie, age, gender, education, health behaviors, disease burden, health literacy, eHealth literacy, and wearable use) are associated with the perceived effectiveness of health apps—separately for CVD, diabetes, and both combined.

## Methods

### Sample and Procedure

This study is a secondary analysis of the data of the Pfizer Monitor “App Utilization.” Data were collected in January and February 2018. A population-based sample of 1500 individuals from Germany participated in this Web-based survey. An external and independent polling institute conducted the study (ie, “Gesellschaft für Innovative Marktforschung,” corporation for innovative market research). An invitation to the Web-based questionnaire was sent via email to participants of former surveys.

Participants had to meet the following inclusion criteria: (1) aged ≥35 years and (2) diagnosed with at least 1 of the following diseases, self-reported hypertension, diabetes, stroke, myocardial infarction, and coronary artery disease. Ownership of a mobile device, for example, a smartphone, was not an inclusion criterion.

**Figure 1:**
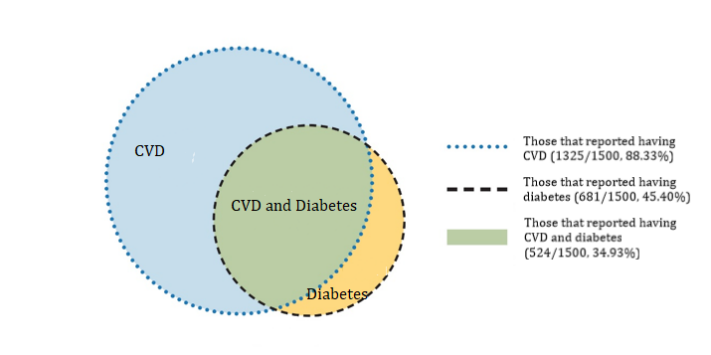
Sample composition and subsamples. CVD: cardiovascular disease.

Participants took an average of 20 min to finish the survey. This study was conducted in compliance with the Declaration of Helsinki; Web-based informed consent was obtained from all participants [46]. An internal ethical and risk assessment was carried out by Pfizer, which approved the Web-based study. For analyses, from the total sample, we selected 3 subgroups: (1) those that reported having CVD (n=1325), (2) those that reported having diabetes (n=681), and (3) those who reported having both CVD and diabetes (n=524; Figure 1).

### Measures

#### Sociodemographics

Sex, age, education (International Standard Classification of Education) [47], occupation, income, and migration background were assessed by standard survey items. Posttax household income by month was categorized as follows: low <€2100, moderate €2100 to €3600, and high >€3600 (1 Euro=US $1.16, August 27, 2018).

#### Cardiovascular Conditions and Diabetes

Cardiovascular conditions and diabetes were assessed by asking participants, “Have you been diagnosed with one or more of the following conditions: (1) CVD, (2) heart failure, (3) coronary artery disease, (4) peripheral artery occlusion disease, (5) myocardial infarction, (6) stroke, (7) hypertension, and (8) atherosclerosis.” Participants were classified as having a CVD if they reported having at least 1 of the conditions. Participants were classified as having diabetes if they reported to have been diagnosed with diabetes. Participants were classified as having diabetes and CVD comorbid if they reported to have diabetes and at least 1 of the other cardiovascular conditions.

Participants were also asked to rate the stress caused by each condition on a scale from 1 (no stress at all) to 5 (very high level of stress). Thus, stress caused by diabetes was assessed with a single item, whereas the overall stress caused only by CVDs was estimated by calculating the mean across the specific present cardiovascular conditions. Assessing the overall stress induced by diabetes and CVDs, the mean stress level across diabetes and all present cardiovascular conditions was calculated.

#### Health Behaviors

Health behaviors were assessed by providing a list of common health-related behaviors (ie, smoking, physical activity, and balanced diet). For smoking, participants were asked, “Do you smoke on a daily basis?” To assess physical activity, participants were asked, “Are you regularly physically active (following World Health Organization, WHO recommendation, ie, 30 min of moderate activity at least 5 times per week or 30 min of intensive activity at least 3 times per week [48])?” Consumption of a balanced diet was measured by asking participants, “Do you follow a balanced diet, that is, eat fruits and vegetables with every meal and including many wholegrain products?”

#### Perceived Health Literacy and Electronic Health Literacy

Health literacy was assessed by the 6-item short-form of the European Health Literacy Survey Questionnaire instrument with a Cronbach alpha of .81 in this study [49]. An example item was the following: “On a scale from very easy to very difficult, how easy would you say it is to find information on treatments of illnesses that concern you?” Answers had a 4-point response format on a Likert scale. eHealth literacy was assessed using the eHealth literacy scale “eHEALS” comprising 10 items [43]. Cronbach alpha was .92 in this study. Example items were as follows: “How useful do you feel the internet is in helping you in making decisions about your health?” and “I know how to use the internet to answer my questions about health.” Answers had a 5-point response format on a Likert scale.

#### App Use

App use was measured by asking participants, “Have you ever downloaded a health app for a smartphone or tablet?” Participants could choose one of the following answers: (1) “Yes, I have and I have used apps recently,” (2) “Yes, I have and I used to use them frequently but not anymore,” (3) “Yes, I have but I don’t use them or just very seldom,” and (4) “No, I have never downloaded a health app.” Participants giving answer (1) or (2) were classified as app users, whereas those giving answer (3) or (4) were classified as nonusers. To assess behaviors targeted by the apps, participants were asked which behavior their most frequently used app targeted.

BCTs of the health apps were chosen in accordance to a taxonomy by Abraham and Michie [50]. In the questionnaire, we provided a list of BCTs, including, for example, *providing information*, *prompting self-monitoring of behavior*, and *prompting specific goal setting.*

To assess the perceived effectiveness of the most frequently used app on participants’ health behavior, the “perceived impact” subscale of the user version of the Mobile Application Rating Scale Questionnaire [51] was used. The perceived effectiveness had a Cronbach alpha of .87 in this survey and a possible scale score ranging from 1 to 5. This scale comprises 66 items, for example, “Intention to change—The app has increased my intentions/motivation to address this health behavior” and “Help seeking—This app would encourage me to seek further help to address the health behavior (if I needed it).”

To access frequency of the use of the most frequently used app, patients were asked, “How often do you use this health app?” Possible answers were “less than once a month,” “several times a month,” “several times a week,” “daily,” and “several times a day.”

To access duration of the use of the most frequently used app, patients were asked, “When did you start using this health app?” Possible answers were “less than one month ago,” “less than six months ago,” “less than a year ago,” and “more than a year ago.”

The use of wearables was assessed by asking participants, “Which of the following devices do you use?” One of the possible answers was: “A wearable/tracking watch”.

### Statistical Analyses

We analyzed the total sample (N=1500) as well as disease-specific subgroups, that is, (1) individuals reporting having at least CVD (N=1325), (2) at least diabetes (N=681), and (3) both CVD and diabetes combined (N=524; Figure 1). Descriptive sample characteristics were provided both for the total sample and the sample of app users except for the app-related variables, which were provided only for app users. Binary logistic regressions were conducted, and 3 parallel models with app use as outcome were calculated: individuals with CVD (model 1), individuals with diabetes (model 2), and individuals with CVD and diabetes (model 3). Covariates were age, gender, health behaviors (ie, smoking, physical activity, and balanced diet), educational level, health literacy, and eHealth literacy. Further covariates were the presence of diabetes and stress by CVD in model 1, the presence of CVD and stress by diabetes in model 2, as well as stress by both disease groups in model 3.

Finally, we applied linear regression analyses to estimate associations with the perceived effectiveness of mobile health apps in the total sample of app users (n=402). The following covariates were used in the model: age, gender, health behaviors, health literacy, eHealth literacy, stress caused by all present diseases including CVD and diabetes, the presence of diabetes, the presence of CVD, the presence of both CVD and diabetes comorbid, frequency and duration of app use, and the number of BCTs.

## Results

### Characterization of the Sample

A total of 1500 individuals completed this population-based Web-based survey (see Table 1). The mean age was 55.10 (SD 8.25) years and 56.53% (848/1500) were women. In terms of education, 70.60% (1059/1500) had a vocational qualification, 24.60% (369/1500) had a university degree, and 4.80% (72/1500) had basic qualification or none. Most participants were working full-time (622/1500, 41.67%) and had a medium household income (591/1500, 39.40%). A minority of 7.53% (113/1500) had a migration background.

Although we only included participants in the study with CVD and diabetes, the most commonly reported chronic conditions among the participants were hypertension (1224/1500, 81.60%) and diabetes (681/1500, 45.40%). Participants rated the overall stress caused by their chronic conditions, that is, CVD and diabetes, 2.64 out of 5 (SD 0.91). Concerning health behaviors, half of the sample reported to be engaged in regular physical activity (751/1500, 50.07%). Furthermore, most participants reported consuming a balanced diet (1074/1500, 71.60%), whereas every third individual was a smoker (553/1500, 36.87%). The mean health literacy was 2.76 out of 5 (SD 0.49), and the mean eHealth literacy was 3.68 out of 5 (SD 0.73).

In total, 87.27% (1309/1500) of the participants owned a smartphone, out of which, 29.49% (386/1309) used health apps. Overall, 26.80% (402/1500) of the participants were classified as health app users (Table 2). Among participants with CVD, 25.41% (339/1334) were mobile health app users, with 29.2% (199/681) of diabetic participants and 27.6% (146/529) of CVD and diabetic participants reporting app use. The most common behaviors that used apps targeted were physical activity (289/402, 71.9%), weight loss (150/402, 37.3%), and nutrition (146/402, 36.3%). The following BCTs were most frequently included within apps: Prompting self-monitoring of behavior (236/402, 58.7%), prompting specific goal setting (224/402, 55.7%), and providing feedback on performance (199/402, 49.5%). Less than 10% of the participants rated that the apps did not contain any BCT (34/402, 8.5%). A quarter of participants used their apps once a day (104/402, 25.9%) and 20.4% (82/402) reported app use several times a day (82/402, 20.4%). The duration of app use was more than a year for 38.1% (n=153/402) of participants, whereas 10.7% (43/402) started less than a month ago. App users rated the perceived effectiveness of the apps on their health 3.79 out of 5 (SD 0.73).

**Table 1:** Sample characteristics by subgroups (N=1500).

Item		Total sample (n=1500)	Participants diagnosed with CVD^a^ (n=1334)	Participants diagnosed with diabetes (n=681)	Participants diagnosed with CVD and diabetes (n=529)	App users of the total sample (n=402)
Gender (men), n (%)		848 (56.53)	568 (42.58)	277 (40.7	200 (37.8)	251 (62.4)
Age (years), mean (SD)		55.10 (8.25)	55.47 (8.00)	54.91 (8.67)	55.68 (8.25)	51.61 (9.52)
**Educational level (International Standard Classification of Education), n (%)**						
	No or basic qualification	72 (4.80)	60 (4.50)	36 (5.3)	26 (4.9)	4 (1.0)
	Vocational qualification	1059 (70.60)	945 (70.84)	481 (70.6)	376 (71.1)	261 (64.9)
	University degree	369 (24.60)	329 (24.66)	164 (24.1)	127 (24.0)	137 (34.1)
**Occupational status, n (%)**						
	Working full-time	622 (41.67)	537 (40.25)	273 (40.1)	198 (37.4)	242 (60.0)
	Working part-time	220 (14.67)	196 (14.69)	84 (12.3)	61 (11.5)	46 (11.4)
	Not working	100 (6.67)	89 (6.67)	45 (6.6)	36 (6.8)	12 (3.0)
	Retired	440 (29.33)	402 (30.13)	226 (33.2)	189 (35.7)	77 (19.2)
	In school	6 (0.40)	6 (.45)	5 (0.7)	5 (1.0)	4 (1.0)
	Other	112 (7.47)	104 (7.80)	48 (7.1)	40 (7.6)	21 (5.2)
**Monthly posttax household income^b^** **, n (%)**						
	Low	513 (34.20)	453 (33.96)	248 (36.4)	196 (37.1)	94 (23.4)
	Medium	591(39.40)	528 (39.58)	266 (39.1)	206 (38.9)	173 (43.0)
	High	277 (18.47)	250 (18.74)	75 (11.0)	93 (17.6)	115 (28.6)
	No answer	119 (7.93)	103 (7.72)	49 (7.2)	34 (6.4)	20 (5.0)
	Migration background, n (%)	113 (7.53)	92 (6.90)	60 (8.8)	43 (8.1)	53 (13.2)
**Chronic conditions, n (%)**						
	Heart failure	236 (15.73)	236 (17.69)	97 (14.2)	97 (18.3)	71 (17.7)
	Coronary artery disease	259 (17.27)	259 (19.42)	95 (15.0)	95 (18.0)	79 (19.7)
	Peripheral artery occlusion disease	678 (45.20)	146 (10.94)	95 (14.0)	74 (14.0)	40 (10.0)
	Myocardial infarction	146 (9.73)	156 (11.69)	52 (7.6)	52 (9.8)	49 (12.2)
	Stroke	156 (10.40)	141 (10.57)	40 (5.9)	40 (7.6)	33 (8.2)
	Hypertension	1224 (81.60)	1224 (91.75)	487 (71.5)	487 (92.1)	306 (76.1)
	Atherosclerosis	243 (16.20	243 (18.22)	97 (14.2)	97 (18.3)	64 (15.9)
	Stress by CVD+diabetes, mean (SD)	2.64 (0.91)	2.63 (0.91)	2.68 (0.88)	2.69 (0.87)	2.84 (0.73)
	Stress by CVD, mean (SD)	2.58 (0.94)	2.58 (0.94)	2.55 (0.95)	2.55 (0.95)	2.76 (0.91)
	Stress by diabetes, mean (SD)	2.78 (1.02)	2.81 (1.04)	2.78 (1.02)	2.81 (1.04)	3.10 (0.97)
**Health behaviors, n (%)**						
	Smoking	553 (36.87)	490 (36.73)	256 (37.6)	196 (37.1)	142 (35.3)
	Physical activity	751 (50.07)	661 (49.55)	326 (47.9)	244 (46.1)	271 (67.4)
	Balanced diet	1074 (71.60)	947 (70.99)	516 (75.8)	395 (74.7)	310 (77.1)
	Health literacy, mean (SD)	2.76 (0.49)	2.75 (0.49)	2.77 (0.48)	2.74 (0.48)	2.88 (0.51)
	Electronic health literacy, mean (SD)	3.68 (0.73)	3.68 (0.72)	3.65 (0.76)	3.64 (0.48)	4.01 (0.59)
	App use, n (%)	402 (26.80)	339 (25.41)	199 (29.2)	146 (27.6)	402 (100)

^a^CVD: cardiovascular disease.

^b^Posttax household income: Low <€2100, moderate €2100-€3600, high >€3600 (1 Euro=US $1.2; May 30, 2018).

**Table 2:** Characteristics of health apps and health app use.

Item		Statistics
App use, n (%)		402 (100)
Perceived effectiveness, mean (SD)		3.79 (0.73)
**Frequency of app use, n (%)**		
	<once a month	22 (5.5)
	Several times a month	79 (19.7)
	Several times a week	115 (28.6)
	Once a day	104 (25.9)
	Several times a day	82 (20.4)
**Duration of app use, n (%)**		
	<1 month	43 (10.7)
	<6 months	116 (28.9)
	<1 year	90 (22.4)
	>1 year	153 (38.1)
**Behaviors targeted by the apps, n (%)**		
	Physical activity	289 (71.9)
	Nutrition	146 (36.3)
	Weight loss	150 (37.3)
	Measuring, for example, blood pressure, blood sugar, and step counter	184 (45.8)
	Sleep control	123 (30.6)
	See patient’s chart or labs	21 (5.2)
	Relaxation	30 (7.5)
	Records on disease	61 (15.2)
	Stop health detrimental behavior	16 (4.0)
	Contact doctor	23 (5.7)
	Medication adherence	34 (8.5)
	Health information	28 (7.0)
	Other	10 (2.5)
**Behavior change techniques, n (%)**		
	Providing information	101 (25.1)
	Prompting self-monitoring of behavior	236 (58.7)
	Prompting barrier identification	33 (8.2)
	Prompting specific goal setting	224 (55.7)
	Providing instruction	108 (26.9)
	Providing feedback on performance	199 (49.5)
	Providing instruction	58 (14.4)
	Providing opportunities for social comparison	54 (13.4)
	Planning social support	32 (8.0)
	Relapse prevention	23 (5.7)
	Training Emotional control	37 (9.2)
	No BCT	34 (8.5)
	Wearables used routinely	97 (24.1)

**Table 3:** Multivariate associations with app use.

Covariate		App use in CVD^a,b^ (N=1325)		App use in diabetes^c^ (N=681)		App use in CVD and diabetes combined^d^ (N=524)	
		Odds ratio (95% CI)	*P* value	Odds ratio (95% CI)	*P* value	Odds ratio (95% CI)	*P* value
Intercept		0.02^e^	<.001	0.01^e^	<.001	0.02^e^	.004
Age		0.93 (0.91-0.95)	<.001	0.94 (0.92-0.97)	<.001	0.93 (0.91-0.96)	<.001
Gender (men vs women)		0.68 (0.50-0.94)	.02	0.64 (0.42-0.98)	.04	0.70 (0.42-1.17)	.17
**Health behaviors**							
	Smoking	0.84 (0.61-1.16)	.30	0.99 (0.66-1.49)	.95	0.89 (0.55-1.44)	.64
	Physical activity	1.78 (1.30-2.43)	<.001	2.12 (1.40-3.20)	<.001	2.16 (1.34-3.47)	.002
	Balanced diet	1.18 (0.83-1.69)	.35	1.31 (0.79-2.18)	.30	1.54 (0.85-2.80)	.16
**Education**							
	No or basic qualification	Ref^f^	Ref	Ref	Ref	Ref	Ref
	Vocational qualification	6.00 (1.71-31.03)	.005	2.90 (0.91-9.22)	.07	2.62 (0.65-10.48)	.18
	University degree	8.38 (2.34-30.07)	.001	3.53 (1.06-11.75)	.04	3.59 (0.84-15.24)	.08
	Health literacy	1.10 (0.79-1.53)	.58	1.22 (0.79-1.89)	.37	1.47 (0.88-2.46)	.14
	Electronic health literacy	2.52 (1.94-3.28)	<.001	2.36 (1.69-3.29)	<.001	2.23 (1.50-3.31)	<.001
	CVD	—^g^	—	0.88 (0.54-1.42)	.59	—	—
	Diabetes	1.52 (1.12-2.06)	.008	—	—	—	—
	Stress by CVD+diabetes	—	—	—	—	1.55 (1.17-2.04)	.002
	Stress by CVD	1.29 (1.09-1.51)	<.001	—	—	—	—
	Stress by diabetes	—	—	1.51 (1.23-1.85)	<.001	—	—
	Wearable use	21.44 (11.60-39.63)	<.001	12.64 (5.48-29.12)	<.001	16.88 (5.92-48.14)	<.001

^a^CVD: cardiovascular disease.

^b^In this model, Nagelkerke R^2^=.391.

^c^In this model, Nagelkerke R^2^=.380.

^d^In this model, Nagelkerke R^2^=.395.

^e^Missing data: CI.

^f^Ref: reference category set to 1.

^g^Not integrated in this model.

### What Factors Are Associated With App Use?

Table 3 displays multivariate associations of app use for all 3 cohorts.

#### Participants Diagnosed With Cardiovascular Disease

Results from a binary logistic regression revealed that among people classified as having CVD (N=1325), app users were significantly younger (odds ratio, OR 0.93; *P*<.001) than nonusers (see Table 3). Furthermore, women used apps more frequently than men (OR 0.68, *P*=.02). App users more often report to meet the WHO norms for physical activity (OR 1.78, *P*<.001), and they reported a higher level of education than nonusers—ie, people with vocational qualification (OR 6.00, *P*=.005) and university degree (OR 8.38, *P*=.001) were more engaged in app use than participants with no or basic qualification. In addition, compared with nonusers, app users had higher eHealth literacy (OR 2.52, *P*<.001). Participants with diabetes as comorbidity were more likely to use health apps than those without having diabetes, (OR 1.52, *P*=.008). Furthermore, app users reported being more affected by their cardiovascular condition (OR 1.29, *P*<.001) than those who were not using apps. Finally, app use was strongly associated with the ownership of wearables (OR 21.44, *P*<.001). There was no association of app use with smoking, balanced diet, and health literacy.

#### Participants Diagnosed With Diabetes

Among participants classified as having diabetes (N=681), health app users were younger (OR 0.94, *P*<.001), more likely to be female (OR 0.64, *P*=.04), reported a higher level of education as people with university degree, and more engaged in app use than those participants with basic qualifications or none (OR 3.53, *P*=.04). Moreover, health app users were more likely to be physically active (OR 2.12, *P*<.001) than nonusers, had higher levels of eHealth literacy (OR 2.36, *P*<.001), and reported being more affected by diabetes (OR 1.51, *P*<.001). Finally, app users were more likely to own wearables than nonusers (OR 12.64, *P*<.001). There was no association of app use with smoking, balanced diet, health literacy, and the presence of CVD.

#### Participants Diagnosed With Cardiovascular Disease and Diabetes

App users classified as having diabetes and CVD (N=524) were younger (OR 0.93, *P*<.001) and more likely to be physically active (OR 2.16, *P*=.002) than nonusers. Furthermore, app users had a higher level of eHealth literacy (OR 2.23, *P*<.001), were more affected by their diseases (OR 1.55, *P*=.002), and were more likely to use wearables (OR 16.88, *P*<.001). There was no association of app use with gender, smoking, balanced diet, education, and health literacy.

### What Factors Are Associated With the Perceived Effectiveness of an App?

Among all app users, those who were younger (B 1.47, *P*=.006) were more likely to report that their apps had a positive effect on the targeted health behavior (see Table 4). Furthermore, participants with higher health literacy (B .24, *P*<.001) and eHealth literacy (B .47, *P*<.001), as well as those who were more affected by their present diseases (B .08, *P*=.04) perceived their app as more effective on their health behavior. Finally, those apps that were reported to contain more BCTs (B .05, *P*=.002) had a greater perceived effect on users’ health behaviors. There was no association of the perceived effectiveness of an app with gender, health behaviors, education, CVD and diabetes, frequency and duration of health app use, and the use of wearables.

**Table 4:** Multivariate associations with the perceived effectiveness of the apps in all app users (N=402).

Item		Perceived effectiveness on health behavior^a^		
		B^b^	95% CI	*P* value
Intercept		1.45	0.51-2.39	.003
Age		−.01	−0.02 to 0.00	.007
Gender (men vs women)		−.05	−0.18 to 0.08	.46
**Health behaviors**				
	Smoking	.07	−0.06 to 0.20	.30
	Physical activity	.10	−0.04 to 0.24	.16
	Balanced diet	.12	−0.03 to 0.28	.11
**Education**				
	No or basic qualification	Ref^c^	—^d^	—
	Vocational qualification	−.36	−0.97 to 0.25	.25
	University degree	−.48	−1.09 to 0.14	.13
	Health literacy	.24	0.11-0.38	<.001
	Electronic health literacy	.47	0.35-0.59	<.001
**Conditions**				
	Diabetes	Ref	—	—
	CVD^e^	−.10	−0.29 to 0.09	.29
	Comorbid CVD and diabetes	−.02	−0.16 to 0.12	.76
	Stress by CVD+diabetes	.08	0.00-0.15	.04
	Frequency of app use	.05	−0.01 to 0.10	.11
	Duration of app use	.02	−0.04 to 0.08	.52
	Number of behavior change techniques	.06	0.02-0.09	.002
	Wearable use	−.05	−0.20 to 0.10	.49

^a^In this model, R^2^=.345.

^b^Unstandardized coefficient B.

^c^Ref: reference category.

^d^Not applicable.

^e^CVD: cardiovascular disease.

## Discussion

### Principal Findings

This study aimed to investigate associations of health literacy, eHealth literacy, and sociodemographic factors with health app use in 3 distinct samples of patients: those with CVD, those with diabetes, and those with CVD and diabetes combined. Furthermore, we aimed to detect relationships between these factors and the perceived effectiveness in patients who used health apps. Across subsamples, we found that every fourth participant reported using apps for health-related purposes. In general, the association patterns were largely comparable across groups with CVD, diabetes, or both conditions. Across conditions, health app users were younger, more likely to be female (apart from those with comorbid CVD and diabetes), better educated, and tended to be physically active. App users had higher eHealth literacy and tended to be more affected by their condition than nonusers. Health literacy was not significantly associated with app use in all 3 condition subgroups. Mobile app users reporting a higher effectiveness of apps on their health behavior tended to be younger, more health literate, and more eHealth literate. Furthermore, they were perceived a stronger burden by their diseases. Apps that were reported as including more BCTs had a higher perceived effectiveness. Finally, the use of wearables was strongly related with health app use; however, wearables were not associated with the perceived effectiveness of health apps.

### Strengths and Limitations

This survey was one of the first nationwide surveys in Germany focusing on mobile health app use. As only people with CVD or diabetes were included in the survey, we were able to examine different subgroups, including those with CVD and diabetes, which represent relevant chronic conditions in terms of prevalence, patient burden, and health care costs. We made use of validated scales such as for health literacy, eHealth literacy, and the perceived effectiveness of the apps. A limitation includes the use of perceived effectiveness rather than measures of behavioral or health outcomes in this study. Nonetheless, as CVD and diabetes are chronic conditions, which have to be treated over decades, it is very important to foster a patient’s (1) awareness, (2) knowledge, (3) attitudes, (4) intention to change, (5) help seeking, and (6) behavior change. These facets are ingredients of the perceived effectiveness score used in the survey. Furthermore, perceived effectiveness has been shown to be a relevant predictor of app use and purchasing decision and outcome satisfaction [52,53]. In addition, perceived effectiveness has been shown to be related with health behavior and adherence [54]. Another limitation is related with the self-report measure of BCTs. Self-report of BCTs used by the health apps might not be a reliable measure as it might be difficult for the participants to identify specific BCTs. However, participants had the opportunity to look into their devices while filling out our questionnaires to gain more accurate reports. Moreover, BCTs that have been used frequently may be recalled more reliably than BCTs that were not used frequently. The parsimonious BCT assessment is another advantage, although future studies should validate users’ self-reports with external BCT ratings. A further limitation was that the cross-sectional design and causation cannot be inferred. Thus, the novel associations found in this study need to be replicated in longitudinal and experimental studies. Finally, we used a brief self-report measure for health behaviors. Although this parsimonious measure is suitable for large-scale surveys, its overreporting of physical activity and underreporting of diet is common, and our findings need to be treated as a first approximation. The sample seems to be more physically active and eats healthier than the average population, although our sample is a clinical one that is likely to be different from the general population; therefore, it cannot be compared with the general population. Nonetheless, it is likely that health behaviors were overreported to a degree in this study.

### Health App Use

The extent of health app use found in this study is comparable with those in the literature among general population samples [23,55]. Furthermore, we found age-related disparities in the use of health apps, which has been shown by previous research in the general population [23,25,27]. In our survey, higher eHealth literacy was associated with higher app use, whereas health literacy was not associated with app use. Previous surveys that did not differentiate between health literacy and eHealth literacy have shown a correlation between higher health literacy and app use [23,25,27]. In their survey, Cho et al examined the role of eHealth literacy on app use in a sample of 765 participants in South Korea [44]. In contrast to their previous expectations, they found that eHealth literacy did not have a direct effect on app use, but the association was mediated by health app use efficacy. In our survey, health app use efficacy was not measured; therefore, it might be possible that we missed out this mediating effect. Future studies should consider health app use efficacy as a relevant factor.

We found that women are more likely to use health apps compared with men. A possible explanation could be that women might care more about a healthy lifestyle [56,57]. Some studies showed that women were more often health app users [58], whereas others showed no sex difference [23,27]. Interestingly, sex was not associated with health app use in participants who reported having diabetes and CVD combined. It can be hypothesized that men diagnosed with multiple conditions care more about a healthy lifestyle than those with single conditions. This could balance the lead of women’s awareness. More research is needed to understand this finding. Another difference in the analyses of the subgroups was that app users in the CVD subgroup tended to have diabetes as comorbidity, whereas diabetic app users did not tend to have CVD as comorbidity. A possible explanation is that the presence of diabetes is driving the intention to use health apps rather than the presence of CVDs, which tend to be less homogeneous in the symptoms and tend to be often asymptomatic, for example, in the case of hypertension [8].

For CVD patients, strategies such as raising awareness of asymptomatic phases of their disease are needed. Although we did not aim to compare the subgroups directly, overall, there were no considerable differences among the subgroups with CVD, diabetes, and CVD and diabetes combined. However, as Figure 1 displays, these 3 subgroups largely overlap and thus cannot be considered as independent groups. A direct comparison among these overlapping groups is not possible. Nonetheless, providing separate estimates for these may guide practitioners and policy makers who are particularly interested in 1 of these subgroups. Furthermore, in terms of comorbidity and multimorbidity, our findings show that regardless of the specific condition, health apps may target shared risk factors such as physical inactivity, nutrition, and weight control, which may be beneficial for all 3 subgroups. Those with both CVD and diabetes may especially benefit, as a reduction of risk factors may result in relief of symptoms of both conditions. Finally, eHealth literacy was superior to health literacy in relation with app use across subgroups, which should be considered in interventions. Thus, interventions are needed that are specific to digital literacy and may be independent from general health literacy.

### Perceived Effectiveness of Health Apps

Younger participants rated their apps to be more effective, in line with previous literature [34,44]. A possible explanation could be a burden of applicability and implementation among older people. Other associations of perceived effectiveness found in this study were health literacy and eHealth literacy, with better health literacy and eHealth literacy being associated with higher perceived effectiveness. People need high competences to adopt health apps effectively. As the importance of health apps is growing, it is necessary to improve people’s health literacy competences. Another option to increase the effectiveness could be to improve the usability of health apps so that even people with limited health literacy might take advantage of health apps. We did not find that frequent health app use was associated with perceived effectiveness contrary to a recent systematic review that suggested that higher use of health apps was associated with an increase in actual health behavior [59]. It might be possible that people use apps for different reasons than improvement of health behaviors, for example, to connect with friends or for fun and enjoyment [60]. The association between app use and perceived effectiveness should be further examined in future research. Concerning properties of the apps themselves, we found that the more BCTs people perceived to be present, the superior was their effect on people’s health. In general, the utilization of BCTs in interventions is a relevant factor for successful behavior change [50]. The integration of BCTs in interventions leads to better outcome in health apps as well [38,61]. For example, the most powerful BCT found in a systematic review was “stress management” or “general communication skills training” [37]. For health apps, planning and monitoring have been found to be BCTs that are related with higher extent of physical activity [23].

### Implications for Further Research, Policy, and Practice

According to the WHO, 121 countries have already developed a national eHealth strategy [62]. Governments should consider that many people still have to face barriers in the use of mobile health apps [63]. Age- and literacy-related disparities should be taken into account. In our research, we found that health literacy and eHealth literacy are linked to superior effects of the apps. This might implicate that it is necessary to increase people’s literacy competences. A possibility could be to integrate health literacy interventions within the apps to foster people’s ability to adequately use apps. Moreover, we found eHealth literacy to be superiorly associated with app use compared with perceived health literacy, which has implications for practitioners and policy makers who aim to improve app use of the diabetes and CVD patient group. Following this new finding, specific interventions should directly target eHealth literacy beyond more general components of health literacy. Moreover, eHealth literacy should be established as an indicator of app use competencies in future app studies as well as in health monitoring of the population.

The current trends in mobile phone use indicate that older people are increasingly engaged in the use of mobile technologies [64]. It can be assumed that in the future, more people will use health apps as the youth grow older. This underlines the potential of mobile health in the future [65]. New ways of supporting health topics will become more relevant in the future. However, contemporary app developers should keep older people as a target group with special needs in their mind. For example, the design of health apps should be adapted, for example, by applying larger letters and few stimuli. General practitioners should learn more about the possibilities of health apps. Information about health apps could be integrated in doctors’ further training. After such a training, a general practitioner might be more likely to recommend a health app. Furthermore, people with low health literacy should not be forgotten to reduce the “digital gap” between user and nonuser [66]. Low health literacy and eHealth literacy are linked to fewer rates of health app use and lower perceived efficacy of the apps. Better health education should be part of the educational system. Furthermore, about a quarter of those who use health apps also used wearables in this study. However, we did not find an improved perceived effectiveness among those using health apps and wearables combined. Future studies should elaborate on how wearables can contribute to better health monitoring and the possible barriers that may have led to the result in this study. For upcoming research, we recommend objective measures of effectiveness such as a reduced HbA_1c_ values in patients with diabetes and fewer cardiac event in patients with CVD rather than perceived effectiveness. In line of the present findings as well as the scientific evidence, it is indispensable to incorporate BCTs in health apps that have shown to be effective. This ensures efficiency and efficacy.

Our results have further implications for clinical practice. If a doctor wants his chronically ill patients to use health apps, he should have 2 things in his mind. First, not every patient is able to handle apps. Doctors have to select patients according to their literacy skills. Furthermore, doctors may recommend diabetes apps to diabetics, which are more likely to be used. For CVD patients, doctors need other strategies. For example, doctors should raise the awareness of patients especially during the asymptomatic phases of their illness to prevent long-term complications. Second, not every app is suitable to provide appropriate disease management. Especially, patients with CVD and comorbid diabetes are at high risk of developing complications. In this highly vulnerable subgroup, eHealth literacy seems to be a relevant factor of health app use, which is a new finding from this study. However, we observed comparable patterns of app use and perceived effectiveness in those patients diagnosed with CVD, diabetes, and both conditions combined. Thus, clinical recommendations for app use may depend on comparable factors across these specific subgroups of patients. Although patient groups were largely overlapping, medical specialists such as endocrinologists, cardiologists, and general practitioners may be interested in our reporting of the specific patient group of interest. The quality of the apps and the use of theory-based interventions should be ensured [66]. More effective health apps are needed. Governmental recommendations or suggestions from independent institutions that are based on scientific evidence could give the clinical practitioners some orientation.

## Abbreviations

BCT: behavior change technique
CVD: cardiovascular disease
eHealth: electronic health
HbA_1c_: hemoglobin A_1c_
OR: odds ratio
WHO: World Health Organization
